# Supplementary material for: Adjusting the family’s life: A grounded theory of caring for children with special healthcare needs in rural areas, Thailand
Source: PLoS One. 2021 Oct 25;16(10):e0258664. doi: 10.1371/journal.pone.0258664 (PMC8544842; doi:10.1371/journal.pone.0258664)
Supplement: S1 File — (DOCX) [file pone.0258664.s001.docx]

**S1 Semi-structured interview guide.**

1. ‘Can you talk about your experiences of raising the CSHCN when he/she was young’

‘Can you give me for example’.

1. ‘Can you share with me how you dealt with the situation when your child was given diagnosis of his/her or conditions?
2. ‘Can you tell me about the relationship between yourself and the CHSCN ?’

‘How about the CHSCN and siblings or others?’

‘Can you tell me about your experience when you have to care for your other children at the same time?

1. ‘What are the challenges that you faced when caring for the CHSCN’ and ‘can you talk about the opportunities?’

‘When you care for the CSHCN, what impact did this have on you e.g. job, financial, time or to other family member?’

‘What kind of support have you had over the years with your child?’

1. ‘How does your spouse/ grandparents/ child’s sibling/ friends/neighbors/ /communities support you in caring for the CHSCN?’

‘Can you tell me about your feelings when your child was involved with health care providers or was admitted in the hospital?

1. ‘Can you tell me what are yours expectation for their future?’

‘What about your future plan?’

‘If you have a chance to talk with the families who have the similar situation, what are your suggestions?’
