# Supplementary material for: Adjusting the family’s life: A grounded theory of caring for children with special healthcare needs in rural areas, Thailand
Source: PLoS One. 2021 Oct 25;16(10):e0258664. doi: 10.1371/journal.pone.0258664 (PMC8544842; doi:10.1371/journal.pone.0258664)
Supplement: Attachment — Submitted filename: Comments and Recommendation.docx [file pone.0258664.s002.docx]

Dear Editor,

Thank you for inviting me to review manuscript for PLOS ONE entitled "Adjusting the family’s life: A grounded theory of caring for children with special healthcare needs in rural areas, Thailand." (PONE-D-21-08488).

Comments and Recommendation

Children with special health care needs often require care from specialists, typically located in urban tertiary centers.

**Recommendations:**

In Background part

Infectious disease instead of infection disease

In Methods

Forty-three family members of CHSCN participated in in-depth interviews. How many families the proposal studied, meaning those 43 members representing how many families**??? Kindly add to methods.**

The main source of the data that support this research are the interviews carried out from almost all different family members. However, reviewer recommend to highlight and analyze separately care giving mothers, who experience the work of caring for their children on a daily basis , in order to ensure the trustworthiness of the results.

Factors affecting the implementation of digital health programs should be discussed in relation to technological, human, and systems factors connecting patients and their families to the needed care from expert health care providers in particular to families in rural areas.

Reviewer highly recommend authors to highlights home (in persons appointments) or Telehealth Interventions to Teach and Support Parents and Family members especially for children with Autism Spectrum Disorders to reduce their problem behavior.
